# Neutrophil-to-Lymphocyte Ratio as Potential Marker of Outcome After Standard EVAR

**DOI:** 10.3390/diagnostics15212807

**Published:** 2025-11-06

**Authors:** Adriana Toncelli, Federico Filippi, Francesco Andreoli, Giulia Colonna, Claudia Panzano, Roberto Silingardi, Claudio Desantis, Massimo Ruggiero, Maurizio Taurino, Pasqualino Sirignano

**Affiliations:** 1 Vascular and Endovascular Surgery Unit, Department of Clinical and Molecular Medicine, Sant’Andrea Hospital of Rome, Sapienza University of Rome, 00185 Roma, Italy; adriana.toncelli@uniroma1.it (A.T.);; 2 Vascular and Endovascular Surgery Unit, Misericordia Hospital, 58100 Grosseto, Italy; 3 Vascular and Endovascular Surgery Unit, University Hospital of Modena and Reggio Emilia, 00185 Baggiovara, Italy; 4 Vascular and Endovascular Surgery Unit, A. Perrino Hospital, 72100 Brindisi, Italy; claudio.desantis25@gmail.com (C.D.);; 5 Vascular and Endovascular Surgery Unit, Department of General Specialistic Surgery, Sant’Andrea Hospital of Rome, Sapienza University of Rome, 00185 Roma, Italy; pasqualino.sirignano@uniroma1.it

**Keywords:** neutrophil-to-lymphocyte ratio, EVAR, outcomes, systematic inflammation

## Abstract

**Introduction**: The neutrophil–lymphocyte ratio (NLR) has proven to be an inexpensive and easily available inflammatory marker for cardiovascular disease. The aim of the present study is to evaluate a possible association between preoperative NLR value and endovascular aneurysm repair (EVAR) outcomes. **Methods**: A multicentric retrospective study of patients undergoing EVAR in elective setting between 2015 and 2023 was performed. Preoperative NLR was calculated by dividing the absolute neutrophil count by the absolute lymphocyte count, and a cut-off of 5 was used as threshold for the analysis. Primary outcomes (technical success, endograft occlusion, AAA-related reintervention, endoleaks, and mortality rates) were compared between the NLR < 5 and the NLR > 5 group. Kaplan–Meier survival analysis was employed to assess overall survival and the incidence of long-term complications. **Results**: The study initially considered 1360 patients. Eventually, 823 patients were included in the study, of whom 129 (15.7%) with NLR > 5. The latter group presented a higher AAA diameter (59.1 mm vs. 55, mm; *p* = 0.004). Technical success was obtained in 98,9% of all enrolled patients. NLR values were significantly associated with ASA class (*p* = 0.014), involvement of the iliac arteries (*p* = 0.023), duration of ICU stays (*p* < 0.001), and overall length of hospitalization (<0.001). At Kaplan–Meier analysis, patient with NLR > 5 showed a significant lower survival rates (*p* = 0.006), while no significant differences were found in terms of endograft occlusion (*p* = 0.45), AAA-related reintervention (*p* = 0.63), and endoleaks (*p* = 0.49). **Conclusions**: This study highlights the association between the NLR value and an elevated risk of long-term mortality, highlighting its role as an indicator of the patient’s overall clinical condition rather than immediate surgical outcomes. Focusing on this selected group of patients can improve postoperative care and reduce long-term complications.

## 1. Introduction

The role of inflammatory markers in cardiovascular diseases has been extensively investigated, and it is now well established that both neutrophils and lymphocytes play critical roles in the atherosclerotic process, contributing to plaque formation, progression, and destabilization [1]. While the role of white cells counts as a prognostic marker is limited by significant confounders, the neutrophil-to-lymphocyte ratio (NLR), defined as the ratio of absolute neutrophil count to absolute lymphocyte count, has emerged as a useful marker pro-inflammatory state and a reliable indicator of vascular wall inflammatory activity [2], integrating the role of the innate and the adaptive immune system [3]. From a physio-pathological point of view, neutrophil infiltration has been implicated in ischemia–reperfusion injury and hypercoagulable states. It seems that neutrophils contribute more to plaque fissuring and rupture rather than its formation. On the other hand, although lymphocytes are central to plaque development, their role in the plaque instability is marginal, and peripheral lymphopenia has been associated with worse clinical outcomes in CAD [4].

The prognostic role of NLR has been evaluated in patients with coronary artery disease (CAD) undergoing myocardial revascularization, in which it was associated with adverse outcomes and higher mortality [5]. The role of NLR has also been investigated in ischemic stroke, where patients with an NLR greater than 5.67 showed a higher mortality rate. It has been proposed that neutrophil migration into the affected brain region represents the initial response to ischemic injury, typically occurring within 6 to 24 h. Once present in the ischemic and reperfused areas, neutrophils release proteolytic enzymes, such as acid phosphatase and reactive oxygen species, which may contribute to the severity of tissue damage, increase the risk of reinfarction, and lead to poorer neurological outcomes. Regarding vascular diseases, the utility of NLR has been evaluated in patients affected by abdominal aortic aneurysms (AAA) [6], where it was associated with a higher risk of no-shrinkage of the aneurism sac after EVAR, acute limb ischemia, and popliteal aneurysms [7,8]. As an inexpensive and easily accessible marker, NLR could significantly enhance the risk stratification of patients undergoing vascular surgery. However, evidence of its role in predicting long-term outcomes after endovascular aortic repair (EVAR) remains limited.

The primary aim of this multicentric study was to evaluate whether an NLR value greater than 5 could serve as a prognostic predictor in patients treated with EVAR, in terms of overall mortality, endograft patency, the presence of high-flow endoleaks (type I and type III), and the need for reintervention.

## 2. Materials and Methods

A retrospective multicenter analysis was conducted on patients who underwent elective endovascular abdominal aortic aneurysm (AAA) repair using standard endografts. Patients were treated between 2015 and 2023, in the Vascular Surgery Department of Sant’Andrea, University Hospital in Rome, AOU-Policlinico di Modena University Hospital, Misericordia Hospital in Grosseto, and A. Perrino Hospital in Brindisi.

Clinical data were retrospectively collected from medical records and organized in a Microsoft Excel^®^ database (Microsoft Corporation, Redmond, WA, USA). The study adhered to the principles outlined in the Declaration of Helsinki, patients provided consent for procedures, data collection, and analysis.

Indication for AAA repair was based primarily on aneurysm diameter, rate of growth more than 1 cm/years, and aortic wall morphology was also considered, as advised from current European and Italian guidelines. Indications for EVAR or ER were based on age, comorbidities, and patient preferences.

Exclusion criteria included cases of EVAR performed outside the device’s instruction for use (IFU), and urgent cases, as well as symptomatic and/or ruptured AAA. Patients with conditions that could influence the neutrophil-to-lymphocyte ratio (NLR), such as acute infection, recent whole blood transfusion, ongoing immunosuppressive therapy, or corticosteroid use, were excluded from the study. Patients affected by any kind of neoplasia were excluded since this condition has been proven to elevate NLR value [9].

For each patient included in the study, a single preoperative blood sample was collected to measure NLR values. The NLR was calculated as the ratio of neutrophil to lymphocyte counts and was subsequently used in all analyses. Blood counts were collected in test tubes with anticoagulant DTA-K3 and processed through cytometric analysis (Alinity h-series^®^).

### 2.1. Preoperative Work-Out

All patients underwent thorough preoperative assessment, including history and physical examination, anesthesiology and cardiology consultations, electrocardiogram, chest X-ray (two views), and abdominal CT angiography. Preoperative laboratory tests included complete blood count, coagulation profile, liver and kidney function tests, and SARS-CoV-2 swab [10]. Blood samples for the calculation of the neutrophil-to-lymphocyte ratio (NLR) were collected preoperatively, before any endovascular intervention. Patients did not receive treatments that could affect inflammatory markers prior to sample collection, except for standard medical therapy for comorbidities, which was continued according to routine clinical practice.

All measurements (diameter, length, angles) were evaluated using a workstation with dedicated reconstruction software (OsiriX^®^ MD software version 12, PIXMEO, Bernex, Switzerland, and Horos Open Software^®^ version 4 on Mac OS compute) [11]. EVAR procedures were performed with commercially available devices according with their IFU; endograft selection, type of anesthesia, surgical or percutaneous access, and necessity for adjunctive intraoperative procedures (sac embolization and/or patent aortic branches embolization) were tailored for each patient [12].

### 2.2. Follow-Up Protocols

The follow-up protocol included a CT scan within one month postoperatively, duplex ultrasound (DUS) at 3, 6, and 12 months, and annual CT scans thereafter unless otherwise indicated. Follow-up assessments were performed at 1, 6, 12, 24, and 36 months.

### 2.3. Study Outcomes

Enrolled patients were stratified as high- and low-NLR groups in accordance with their preoperative NLR. The threshold value of 5 for the neutrophil-to-lymphocyte ratio (NLR) was chosen based on the existing literature, which has identified this cut-off as clinically significant for risk stratification in patients undergoing vascular procedures. Previous studies have shown that elevated NLR is associated with adverse outcomes, such as 2-year mortality, in patients undergoing elective vascular surgery [13]. Additionally, recent research has confirmed that NLR > 5 predicts 5-year mortality in patients with peripheral arterial disease undergoing femoral endarterectomy [14]. Therefore, adopting a cut-off of 5 in our study is supported by established evidence and allows comparability with previous investigations [7,15].

The primary outcomes were overall mortality, endograft patency, the presence of high-flow endoleaks (type I and type III), and the need for reintervention. Secondary outcomes focused on the relationship between NLR levels and both preoperative factors (AAA diameter, iliac involvement, and comorbidities) and intraoperative factors (ICU stay and length of hospital stay) [16]. All analyses were conducted separately for the two patient groups.

### 2.4. Statistical Analysis

Demographic and clinical data were displayed as mean and standard deviation (SD) for normally distributed data, as median and interquartile range (IQR) for non-parametric data, and as absolute and relative frequencies for the categorical data. The distribution of continuous data was tested using Kolmogorov–Smirnov tests. Categorical data were compared using the Chi square test and continuous variables were compared using independent sample *t*-test or Mann–Whitney U test according to the distribution. Odds ratios (ORs) with 95% confidence intervals (CIs) were calculated to assess the strength of association between NLR and primary outcomes. Kaplan–Meier analysis was used to evaluate survival and complications rates. A *p*-value of <0.05 was defined as statistically significant. All statistical analyses were performed using SPSS, version 27.0 (IBM Corp, Armonk, NY, USA, 2020).

## 3. Results

**Figure 1:**
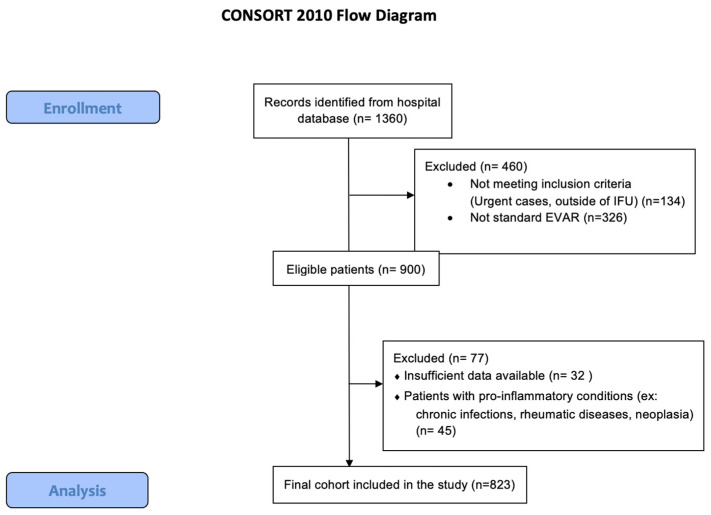
CONSORT DIAGRAM of the presented research.

The study initially considered 1360 patients. For reasons of data completeness and after excluding ineligible cases, 823 patients were enrolled in the study. More details regarding the selection process are presented in Figure 1.

Out of a total of 823 patients, 304 were treated in Rome, 169 in Brindisi, 146 in Grosseto, and 204 in Modena. No statistical differences were found between the center of treatment and the value of NLR (*p* = 0.23).

The mean age of the patients was 75.3 ± 7.9 years, of whom 694 (84.3%) with NLR < 5 and 129 (15.7%) with NLR > 5. There were no notable differences in comorbidities between the groups, except for chronic kidney disease, which was significantly more prevalent in the NLR > 5 group (34.2% vs. 17.4%, *p* < 0.001). The most represented ASA class in the present series was III, with a statistically significantly higher class in the NLR > 5 group (*p* = 0.014). Among patients with NLR > 5, 79.4% presented iliac involvement, showing a significant difference compared with the NLR < 5 group (*p* = 0.023). The distribution of comorbidities, analyzed according to NLR values, is presented in Table 1.

The endovascular grafts employed are the following: AFX/AFX2 (Endologix Inc., Irvine, CA, USA), Anaconda™ AAA Stent Graft System (Terumo Aortic Ltd., Inchinnan, UK), Aorfix™ stent graft (Lombard Medical, Didcot, UK), Zenith Flex^®^/Zenith Alpha AAA Endovascular Graft (Cook Medical Inc., Bloomington, IN, USA), E-tegra™ STENT GRAFT SYSTEM (Jotec GmbH, Hechingen, Germany), Endurant^®^/Endurant II/Endurant IIs AAA Stent Graft System (Medtronic Inc., Dublin, Ireland), GORE^®^ EXCLUDER/C3/CONFORMABLE AAA Endoprosthesis (W. L. Gore & Associates Inc., Flagstaff, AZ, USA), INCRAFT^®^ AAA Stent Graft System (Cordis Corporation, Bridgewater, NJ, USA), Ovation Prime/iX/Alto (Endologix Inc., Irvine, CA, USA), and TREO Abdominal Stent–Graft System (Terumo, Somerset, NJ, USA). More details are shown in Table 2.

Regarding postoperative outcomes, ICU hours (*p* < 0.001) and in-hospital stays (*p* < 0.001) were significantly higher in patients with NLR > 5. Further information can be found in Table 3.

In the present series, overall technical success was achieved in 98.9% of cases. At last follow-up (36 months), 10% (84) of patients died, 3.9% (32) of patients developed high-flow endoleaks, 10.8% of patients had graft occlusions, and 5.6% of patients needed AAA-related reintervention.

When stratified by NLR, 63 patients (75%) who died had an NLR > 5, while 21 patients (25%) had an NLR < 5 (*p* = 0.013, OR 0.51, CI: 0.31–0.87). All other surgical outcomes were not significantly different between NLR > 5 and > 5 groups. More details are shown in Table 4.

**Figure 2:**
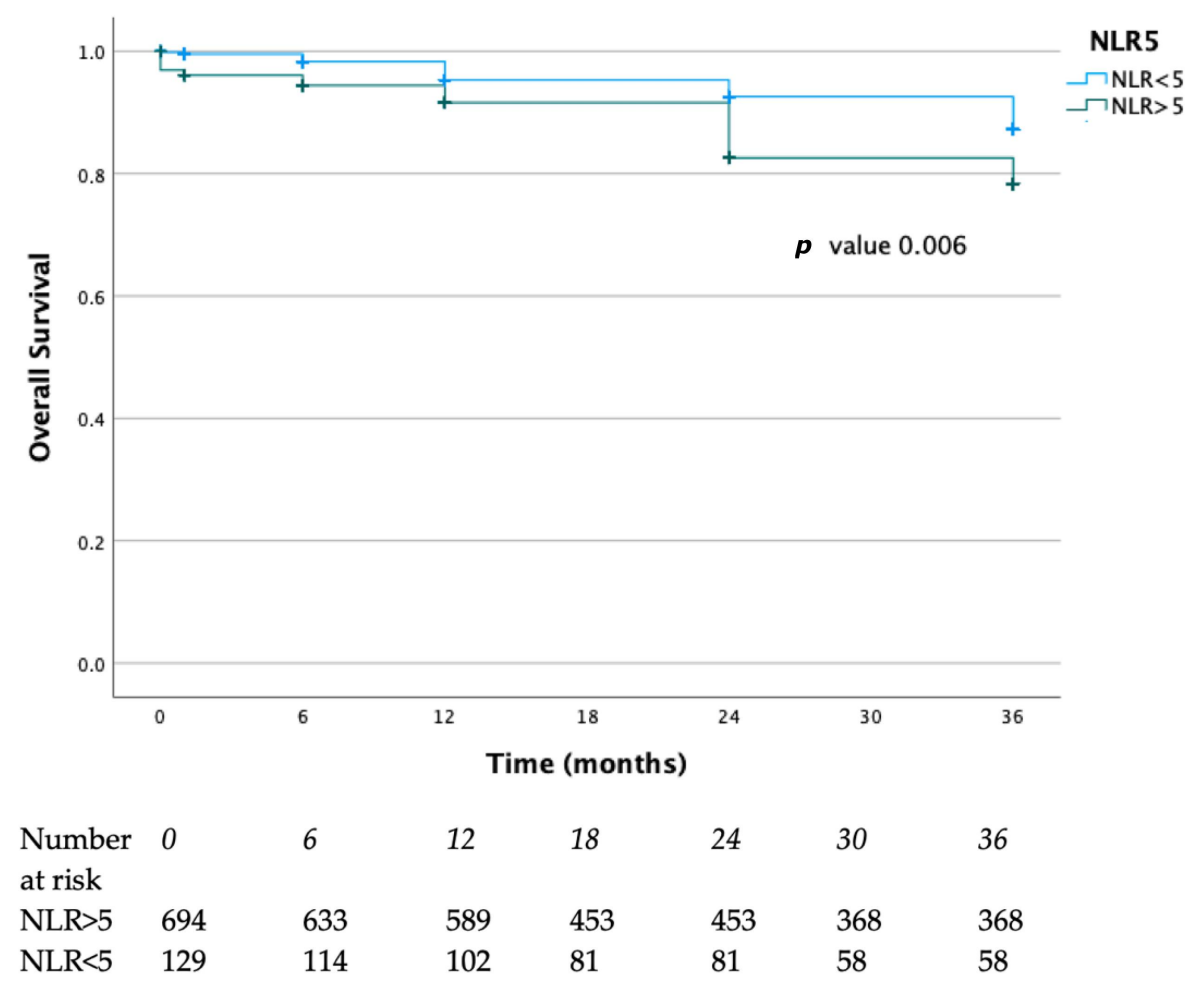
Survival rates of the present case series. Blue lines represent the NLR > 5 group, green lines represent the NLR < 5 group.

At Kaplain–Mayer analysis, the survival rates at 12, 24, and 36 months were 96%, 92%, and 88% in the NLR < 5, and 92%, 80%, and 78% in the NLR > 5, respectively. This difference was statistically significant (*p* = 0.006) (Figure 2).

**Figure 3:**
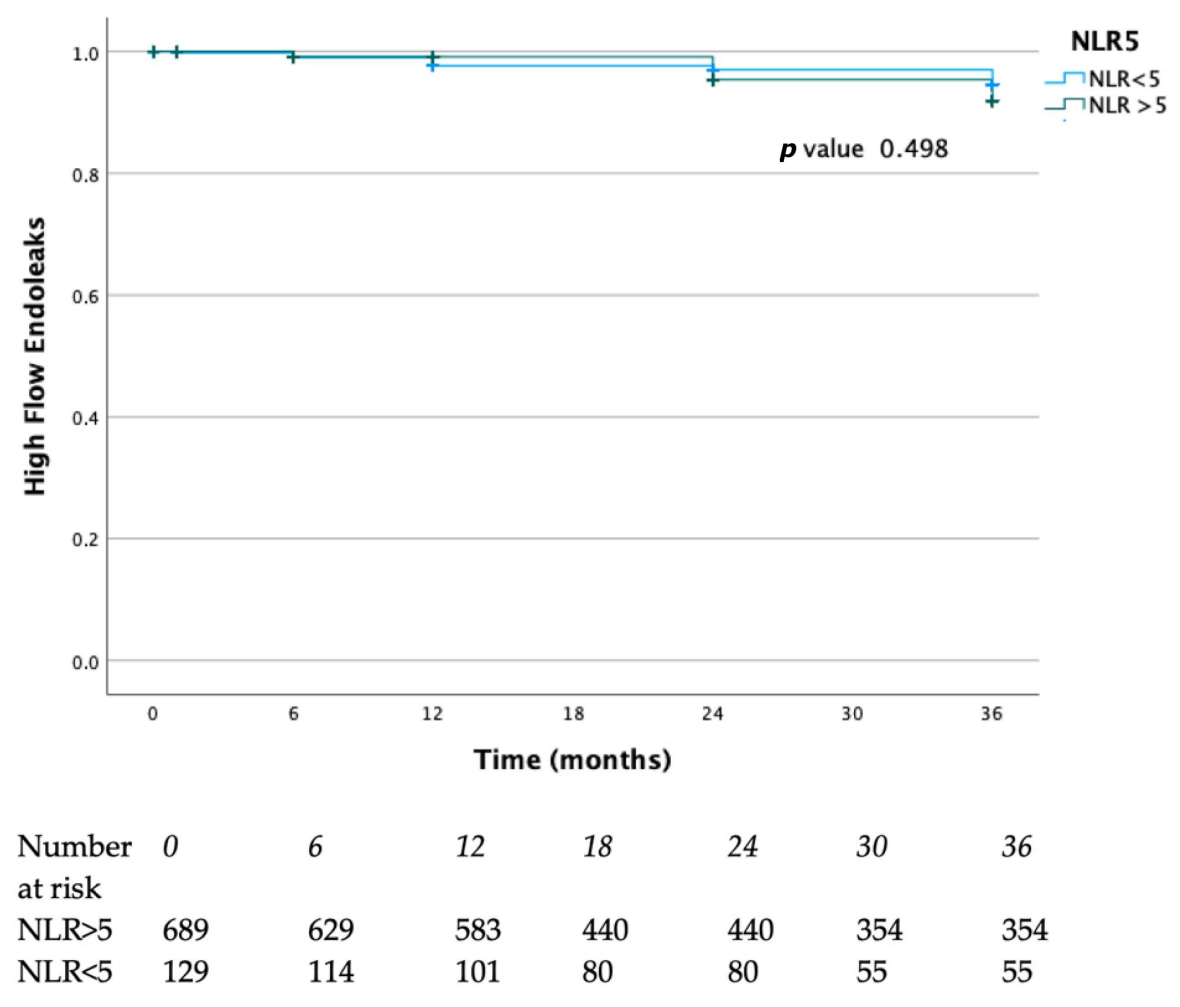
Endoleaks rates of the present case series. Blue lines represent the NLR > 5 group, green lines represent the NLR.

Survival rates free from high flow of endoleaks at 12, 24, and 36 months were 98%, 97%, and 96% in the NLR < 5 group and 99%, 96%, and 90% in the NLR > 5 group, respectively. (*p* = 0.49) (Figure 3).

**Figure 4:**
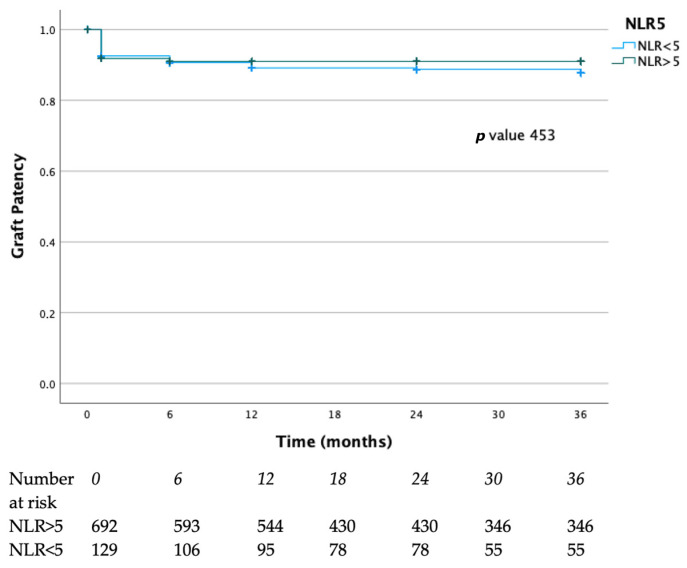
Graft patency rates of the present case series. Blue lines represent the NLR > 5 group, green lines represent the NLR < 5 group.

Graft patency rates at 12, 24, and 36 months were 88%, 87%, and 86% in the NLR < 5 group and 90%, 90%, and 90% in the NLR > 5 group, respectively. (*p* = 0.45) (Figure 4).

**Figure 5:**
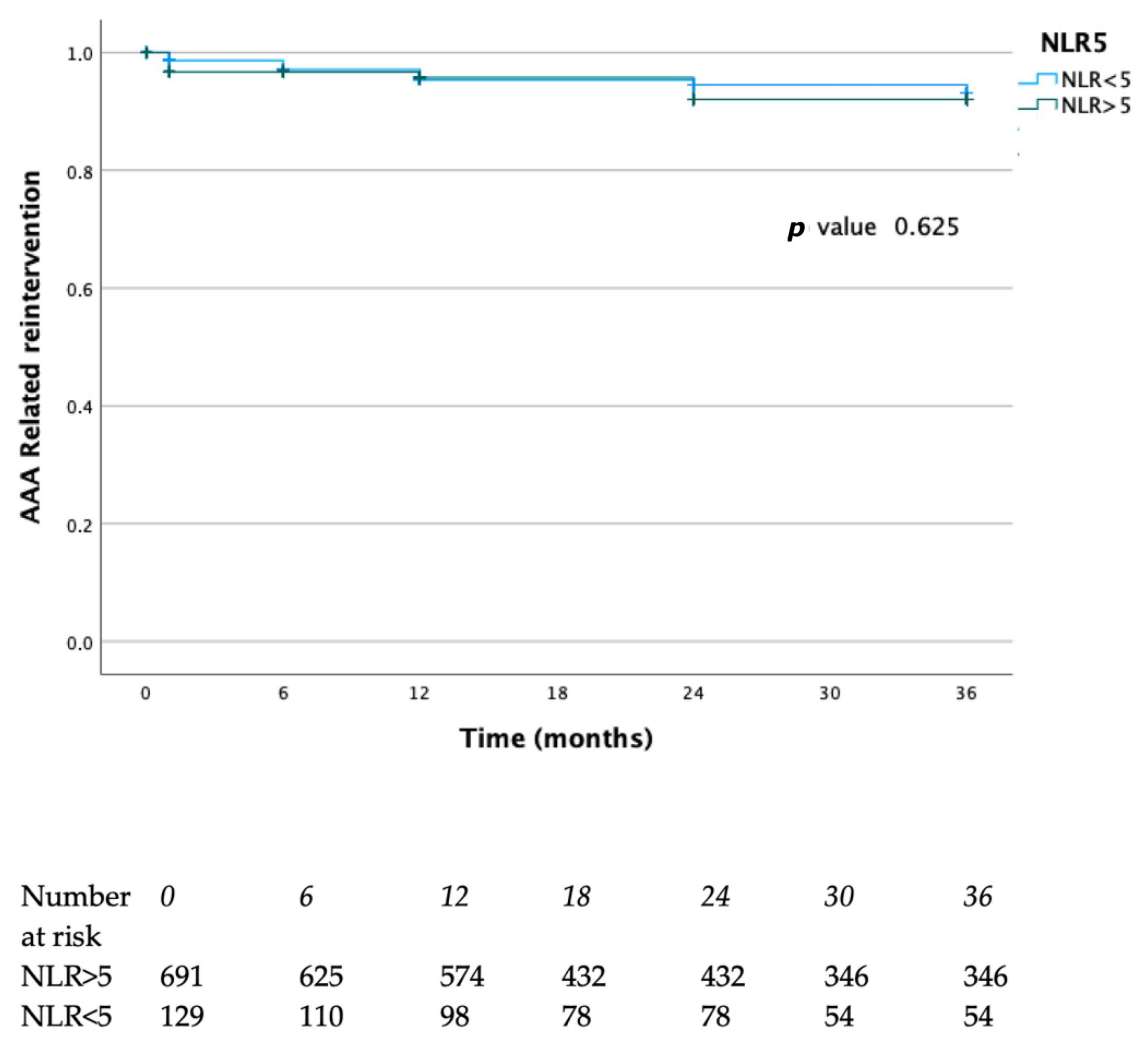
Survival free from AAA-related reintervention rates of the present case series. Blue lines represent the NLR > 5 group, green lines represent the NLR < 5 group.

Survival rates free from reinterventions at 12, 24, and 36 months were 95%, 94%, and 94% in the NLR < 5 group and 95%, 90%, and 90% in the NLR > 5 group, respectively (*p* = 0.62) (Figure 5). Of a total of 84 overall deaths, 8 (9.5%) were related to AAA.

## 4. Discussion

To our knowledge, this is the first study to explore the correlation between NLR and postoperative complication rates following EVAR, showing a significant association between an NLR value > 5 and lower long-term survival rates in patients subjected to EVAR.

Interest in NLR as a prognostic biomarker has significantly increased in recent years. Previous reviews suggested that NLR may offer superior prognostic value compared to absolute leukocyte counts or their subtypes in stratifying cardiovascular risk [1,17]. The potential superiority of NLR could be attributed to its relative stability, being less susceptible to fluctuations induced by physiological stressors such as dehydration or physical exercise. Moreover, NLR reflects the balance between neutrophils, associated with nonspecific systemic inflammation, and lymphopenia, which reflects a general state of psychophysical stress [1]. One of the key advantages of NLR is its simplicity and low cost, particularly when compared to the clinical information it can provide [9].

NLR has been evaluated in several clinical scenarios; in patients with coronary artery disease (CAD), elevated NLR has consistently been associated with poorer long-term outcomes. Several studies demonstrated higher all-cause and cardiovascular mortality in patients with increased NLR following percutaneous or surgical myocardial revascularization [5,18,19].

The neutrophil-to-lymphocyte ratio (NLR) has emerged as a promising biomarker for risk stratification and prognosis in cardiovascular diseases. Recent studies have demonstrated that elevated NLR is associated with an increased risk of both cardiovascular and all-cause mortality across different patient populations. For example, in patients with coronary artery disease and hypertension, an NLR above 2.65 was linked to a significantly higher risk of cardiovascular mortality (HR 1.58) and all-cause mortality (HR 1.46) over a median follow-up of 76 months [20]. Similarly, in a cohort of hypertensive patients, elevated NLR was independently associated with increased cardiovascular (HR 2.33) and all-cause mortality (HR 1.96), with an area under the ROC curve of 0.68 for 10-year cardiovascular mortality [21].

These findings support the prognostic value of NLR and align with the results of our study, where patients with a preoperative NLR > 5 undergoing EVAR showed lower long-term survival. This suggests that NLR may serve as a marker of overall clinical condition rather than predicting immediate procedural outcomes.

In the field of vascular surgery, the NLR has already been investigated across multiple studies. Khanzadeh et al. highlighted that a high NLR value was significantly associated with an increased risk of amputation in patients with acute lower limb ischemia, as well as higher mortality and the occurrence of post-amputation complications [22]. The study conducted by Kordzadeh et al. identified preoperative NLR values > 5 as an independent predictor of 30-day mortality in patients undergoing intervention for abdominal aortic aneurysms (AAAs) [23]. Pasqui et al. reported an association between lower NLR values and aneurysm sac shrinkage, recognizing inflammation as a potential contributor to non-shrinking AAAs [6]. Notably, two studies carried out at our institution have demonstrated that NLR plays a role in patients with acute lower limb ischemia (ALI), and popliteal aneurysms. In both cases, NLR values > 5 were associated with poorer treatment outcomes and increased risk of long-term adverse events [7,15].

In the present study, a value of NLR > 5 was significantly associated with higher mortality rates, both at last follow-up (*p* = 0.013) and in Kaplain–Meier analysis (*p* = 0.006). These findings are consistent with previous research, in which NLR > 5 has been considered an independent predictor of 30-day mortality in patients treated for AAA [13,23]. Moreover, in a recent study conducted by Octeau et al., NLR values were significantly associated with increased 5-year mortality, as well as higher rates of reintervention at 30 days, 1 year, and 5 years [24]. It is worth noting that only 9.5% of deaths were attributed to AAA-related causes, which may indicate that higher NLR values primarily reflect an increased overall clinical frailty and systemic vulnerability of the patients.

This trend was not observed when evaluating other complications such as type I and III endoleaks, aortic graft occlusion, or the need for reintervention, where no statistically significant differences were found between the two NLR groups. Speculatively, the fact that these differences emerged later in the follow-up period suggests that complications in patients with high NLR are more likely related to their overall clinical condition rather than to technical failure of the procedure itself. Conversely, previous research identified an association between low NLR values and persistence at follow-up after type II EVAR [25,26]. The discrepancy between these results and ours could be attributed to differences in study design, patient selection, or follow-up duration. Given the limited data available, further prospective studies are needed to clarify whether NLR influences specific post-EVAR complications or primarily reflects overall health status. However, data regarding the correlation between NLR values and such complications are currently lacking in the literature, and further studies are needed to validate these assumptions.

With regard to secondary outcomes, the present study revealed significant associations between NLR values and ASA class (*p* = 0.014), iliac artery involvement (*p* = 0.023), aneurysm diameter (*p* = 0.004), as well as ICU stay and total length of hospitalization (*p* < 0.001).

The findings regarding secondary outcomes are consistent with growing evidence linking elevated NLR values to greater clinical vulnerability and more complex postoperative courses. Several studies have reported that higher pre- or postoperative NLR correlates with longer hospital stays and increased ICU admission rates after cardiovascular and vascular procedures, supporting our observation that patients with elevated NLR experienced longer ICU stays and overall hospitalization times [26,27,28].

Studies in surgical populations have shown that higher NLR values tend to occur in patients with a greater ASA class or with a greater comorbidity burden, suggesting that NLR reflects a systemic inflammatory and frailty state overlapping with traditional preoperative risk stratification metrics [29]. These data strengthen our interpretation that the observed association between high NLR and higher ASA class indicates overall clinical frailty rather than mere laboratory variability. Although few studies have directly examined iliac artery involvement in relation to NLR in EVAR, research in peripheral artery disease (PAD) suggests that elevated NLR is associated with the extent and severity of atherosclerotic disease, particularly in multilevel or iliac involvement cases [30]. This evidence supports the hypothesis that higher NLR values may correspond to a more advanced and widespread atherosclerotic burden, explaining the greater frequency of iliac extension observed in our high NLR subgroup. Taken together, these results highlight the increased clinical vulnerability of patients with elevated NLR, irrespective of technical procedural success. Therefore, it is safe to assume that NLR may serve as a marker to identify selected groups of patients worthy of a more tailored perioperative management.

This study has several limitations. First, its retrospective design may have introduced selection bias. Second, as a multicenter study, patient management could have varied according to each center’s experience and practices, potentially affecting intraoperative and postoperative outcomes. Third, although patients with known inflammatory conditions that could alter NLR values were excluded, other factors such as medications (ex: acetylsalicylic acid), unconsidered comorbidities, or lifestyle habits may have influenced NLR. In this context, chronic kidney disease, which was more prevalent in the NLR > 5 group, may have contributed to the observed results. Moreover, preoperative C-reactive protein (CRP) levels were not consistently available across all participating centers and therefore were not included in the analysis. The absence of CRP data limits the assessment of possible subclinical inflammatory states that might have influenced NLR values. Future studies should be conducted to elucidate the precise impact of these confounding factors on the stratification of high-risk patients according to NLR values. Additionally, the follow-up in the present study is limited to 36 months. Finally, although the present study focused specifically on the prognostic value of NLR in patients undergoing EVAR, it would be valuable in future research to compare NLR with other established biomarkers to further refine risk stratification and improve predictive accuracy. This represents an important area for further investigation. Further research is warranted to assess the long-term outcomes in this selected patient population.

## 5. Conclusions

The present study demonstrates that elevated neutrophil-to-lymphocyte ratio (NLR) values are associated with an increased risk of long-term mortality, though not with other complications. These findings support the potential utility of NLR as a predictive marker of the overall clinical status of patients subjected to EVAR, and emphasize the need for a tailored approach for this selected group of patients in order to improve postoperative management and reduce long-term complications.

## Figures and Tables

**Table 1:** Comorbidities of the patients in the present series (*n* = 823).

	Tot (*N* = 823)	NLR > 5 (*n* = 129)	NLR < 5 (694)	*p* Value
** Male sex **	756 (91.9)	120 (15.9)	636 (84.1)	0.60
**CKD**	152 (18.5)	41 (26.9)	111 (73.1)	<0.001 *
**CAD**	283 (34.4)	47 (16.6)	236 (83.4)	0.594
**DM**	147 (17.9)	25 (17.0)	122 (83.0)	0.624
**HTN**	662 (80.4)	110 (16.6)	552 (83.4)	0.132
**COPD**	281 (34.4)	49 (17.4)	232 (82.6)	0.316
**Smoking**	415 (50.4)	66 (15.9)	349 (84.1)	0.855
**Obesity**	199 (24.3)	23 (11.5)	176 (88.5)	0.061
**Dyslipidemia**	500 (60.8)	75 (15.0)	425 (85.0)	0.508
**Stroke/TIA**	73 (8.9)	11 (15.1)	62 (84.9)	0.881
**ASA Class**				0.014 *
**1**	2 (0.2)	1 (50)	1 (50)	
**2**	320 (38.9)	65 (20.3)	255 (79.3)	
**3**	480 (58.3)	60 (17.5)	420 (87.5)	
**4**	21 (2.6)	3 (14.3)	18 (85.7)	
**Iliac artery involvement**				0.023 *
**Yes**	204 (24.8)	42 (20.6)	162 (79.4)	
**No**	619 (75.2)	86 (13.9)	533 (86.1)	

N: number; CKD: chronic kidney disease; CAD: coronary artery disease; DM: diabetes mellitus; HTN: hypertension; COPD = chronic obstructive pulmonary disease; TIA: transient ischemic attack; preoperatively, the NLR > 5 group resulted in a AAA maximum diameter significantly greater than the NLR > 5 group (59.1 mm vs. 55.7 mm, *p* = 0.004). * the *p*-value is statistically significant.

**Table 2:** Deployed endograft for EVAR in the present series (*n* = 823).

Endograft	*N* (%)
**AFX/AFX2**	172 (20.9)
**Anaconda**	24 (2.9)
**Aorfix**	4 (0.5)
**Zenith**	52 (6.4)
**E-tegra**	16 (1.9)
**Endurant/EndurantII/IISII**	198 (24.0)
**GORE EXCLUDER/C3/Comformable**	274 (33.3)
**INCRAFT**	2 (0.2)
**Ovation Prime/iX/Alto**	52 (6.3)
**TREO**	29 (3.6)

**Table 3:** Postoperative features of the present series.

	Tot (*N* = 823)	NLR > 5 (*N* = 129)	NLR < 5 (*N* = 694)	*p* Value
**Anesthesia**				0.399
General	288 (35.0)	41 (14.2)	247 (85.8)	
Local	535 (65.0)	446 (83.4)	88 (15.6)	
**ICU (h)**	6.7 (+/16.9)	17.4 (±25.8)	4.7 (±4.5)	<0.001 *
**Hospital stays (days)**	5.5 (+/4.6)	6.7 (±4.8)	5.3 (±13.8)	<0.001 *

* the *p*-value is statistically significant

**Table 4:** Surgical outcomes of patients of the present series at last follow-up (36 months).

	Total (*n* = 823)	NLR > 5 N (%) (*n* = 129)	NLR < 5 N (%) (*n* = 694)	*p* Value	Odds Ratio (CI 95%)
Deaths	84 (10.2)	63 (75)	21 (25)	0.013 *	0.51 (0.31–0.87)
Endoleaks	32 (3.9)	6 (18.8)	26 (81.3)	0.63	0.82 (0.32–1.9)
Graft occlusion	89 (10.8)	11 (12.4)	78 (87.6)	0.36	0.73 (0.38–1.4)
Reintervention AAA-related	46 (5.6)	8 (17.4)	38 (84.4)	0.74	0.87 (0.40–1.9)

* the *p*-value is statistically significant

## Data Availability

The raw data supporting the conclusions of this article will be made available by the authors on request.

## Abbreviations

The following abbreviations are used in this manuscript:

AAA	Abdominal Aortic Aneurysm
ALI	Acute Limb Ischemia
ASA	American Society of Anesthesiologists (Classification)
CAD	Coronary Artery Disease
CI	Confidence Interval
CKD	Chronic Kidney Disease
COPD	Chronic Obstructive Pulmonary Disease
CT	Computed Tomography
DM	Diabetes Mellitus
DUS	Duplex Ultrasound
EVAR	Endovascular Aneurysm Repair
HTN	Hypertension
ICU	Intensive Care Unit
IFU	Instructions for Use
IQR	Interquartile Range
NLR	Neutrophil-to-Lymphocyte Ratio
OR	Odds Ratio
SARS-CoV-2	Severe Acute Respiratory Syndrome Coronavirus 2
SD	Standard Deviation
SPSS	Statistical Package for Social Sciences
TIA	Transient Ischemic Attack
